# Gentle interactions with restrained and free-moving cows: Effects on the improvement of the animal-human relationship

**DOI:** 10.1371/journal.pone.0242873

**Published:** 2020-11-23

**Authors:** Annika Lange, Susanne Waiblinger, Anja Heinke, Kerstin Barth, Andreas Futschik, Stephanie Lürzel

**Affiliations:** 1 Department for Farm Animals and Veterinary Public Health, Institute of Animal Welfare Science, University of Veterinary Medicine, Vienna, Vienna, Austria; 2 Institute of Organic Farming, Federal Research Institute for Rural Areas, Forestry and Fisheries, Johann Heinrich von Thünen Institute, Westerau, Germany; 3 Department of Applied Statistics, JK University Linz, Linz, Austria; University of Illinois, UNITED STATES

## Abstract

The animal-human relationship is essential for farm animal welfare and production. Generally, gentle tactile and vocal interactions improve the animal-human relationship in cattle. However, cows that are fearful of humans avoid their close presence and touch; thus, the animal-human relationship first has to be improved to a point where the animals accept stroking before their perception of the interactions and consequently the animal-human relationship can become positive. We tested whether the animal-human relationship of cows fearful of humans is improved more effectively by gentle interactions during restraint, allowing physical contact from the beginning, or if the gentle interactions are offered while the animals are free to move, giving them more control over the situation and thus probably a higher level of agency and a more positive perception of the interactions. Thirty-six dairy cows (median avoidance distance 1.6 m) were assigned to three treatments (each n = 12): gentle vocal and tactile interactions during restraint in the feeding rack (LOCK); gentle vocal and, if possible, tactile interactions while free in the barn (FREE); routine management without additional interactions (CON). Treatments were applied for 3 min per cow on 10 d per fortnight for 6 weeks (i.e., three periods). Avoidance and approach behaviour towards humans was tested before the start of the treatment period, and then at 2-week intervals. The recorded variables were reduced to one score by Principal Component Analysis. The resulting relationship score (higher values implying a better relationship with humans) increased in all groups; the increase was stronger in FREE than in CON, with the increase in LOCK being not significantly different from the other treatment groups. Thus, we recommend that gentle interactions with cows should take place while they are unrestrained, if possible.

## 1 Introduction

Nowadays, there is largely a consensus in the field of animal welfare science that farmed animals should have a good quality of life, not only a life mostly free of aversive experiences [1,2]. This means that the balance of positive and negative emotions should be tipped towards the positive side [1], which requires not only the avoidance of negative emotional states as far as possible, but also the presence of opportunities in the animals’ lives to experience positive emotional states. A good animal-human relationship not only reduces negative emotional states and associated stress, but also provides a possibility for the animals to experience positive emotions, e.g. pleasure during gentle tactile interactions with humans, in addition to being associated positively with good health and production as well as work safety (for reviews, see [3–5]).

An effective method to improve the relationship between cattle and humans is gentle tactile stimulation [6,7], which is thought to mimic social licking, an affiliative behaviour shown by cows [8]. Gentle tactile contact, often combined with talking in a gentle voice, reduces the avoidance distance of cows [9,10] and calves [11,12] and thus improves the animals’ relationship to humans, including a reduction of fear of humans [5,13]. Although gentle tactile interactions are generally effective in improving the animal-human relationship in cattle and seem to be perceived as positive by most of the experimental animals at the end of a treatment period [14–16], a neutral to good quality of the animal’s relationship to humans is necessary as a prerequisite. If the animal-human relationship is poor, animals perceive humans, their close presence and touch as threatening stimuli [5] and animals free to choose will not accept close contact. In order to take advantage of the benefits of a good animal-human relationship mentioned above, it is necessary to improve the perception of the human from negative to neutral and possibly already positive, so that gentle tactile interactions can take place. This happens in the beginning mainly by habituation, first to the human’s presence, decreasing the distance to the animals over time, and probably also to physical contact with the human, so that the animals do not fear the person anymore, resulting in a more neutral perception of humans. At some point, the animals are usually able to enjoy the interactions, and once this point is reached, the improvement of the animal-human relationship can proceed due to positive reinforcement.

Two major approaches to habituation are possible: either the animals are restrained and touched without the possibility to avoid the contact or the animals are free to move. The first approach has the advantage that the animals can learn already from the first day that this contact does not cause them harm. The higher exposure might accelerate the habituation process; then, the cows are able to enjoy the interactions at an earlier time, allowing positive reinforcement and a positive perception of the human. On the other hand, the restraint approach carries the risk that the animals might perceive the treatment initially as aversive due to their fear of humans and lack of possibilities to avoid close contact with them, which might also affect the later perception of the treatment, although we expect it to change quickly to neutral and then to positive. The second approach has the advantage of allowing the animal control over the situation, which should reduce or eliminate the potential stress caused by the approach or presence of a human [17,18] and allow a more positive perception of the interactions. Moreover, recent literature suggests that animal agency in itself can contribute to good welfare [19]. Accordingly, it has been proposed that an experimental stroking treatment might be more effective if the animal plays an active role in the situation [9,16,20]. However, animals with fear of humans cannot be approached closely enough to touch them from the very beginning; first, the relationship to humans has to be improved to a degree that they accept close presence and then touch. The habituation process might thus take longer; the transition from the acceptance of the close presence of a human to a positive perception might occur relatively quickly, though, once the animal can be touched and experiences the positive tactile stimulus. In addition, it might be the case that the effect of the treatment will be more stable over time in the free-moving animals, as the possible occurrence of negative emotions such as fear is minimized.

We tested the hypothesis that the improvement of the animal-human relationship, assessed via avoidance and approach behaviour towards humans, is influenced by the level of control the animal has over the situation. We predicted that the animal-human relationship is improved more quickly in cows that were approached and stroked while restrained (LOCK) than in cows that were able to move freely (FREE) due to a faster process of habituation. However, we expected the animal-human relationship in FREE to improve more strongly (though at a later point in time) due to a more positive perception of the interactions. For the same reason, we expected the positive effect on the animal-human relationship to last longer in FREE than in LOCK after the end of the treatment.

## 2 Methods

### 2.1 Animals, housing and management

The experiment was conducted from January to March 2019 on 36 animals from two herds housed separately on the research farm of the Thünen Institute of Organic Farming, Trenthorst, Germany. Herd 1 consisted of 40 black and white German Holstein cattle (14 with horns, 26 genetically polled). Herd 2 consisted of 44 black and white German Holstein cattle and one German Red Pied (all horned). If necessary for other experiments, the farm rears calves together with their mothers; during the experimental period, there were 5 to 7 calves in both herds. The whole-day contact of dams with their calves during the first three months of lactation and the resulting unlimited suckling is the main reason for the relatively low average milk yield of 6,430 kg/305 days.

The two herds were kept in loose housing, with a roofed lying area consisting of two rows of cubicles (1.24 m x 3 m including headspace) separated by a rubber-floored alley, a partly roofed feeding area and an unroofed alley between the two areas (total space allowance: 785 m^2^ per herd; 17–19 m^2^/animal). The feeding area consisted of two sections accessible via transponder-controlled selection gates only for the respective yield group; for details, see [21]. The cows were milked twice daily (at 05:15h and 15:45h) in a 2x4 tandem milking parlour (GEA Farm Technologies, Bönen, Germany) located between the two compartments; for details, see [10]. A fresh mixed ration for ad libitum consumption was provided twice per day after morning and evening milking. After each milking time, the cows were restrained in the feeding rack approximately until 08:30 h and 18:00 h, respectively, to prevent them from lying down immediately after milking and thus reduce the risk of intra-mammary infections.

**Fig 1:**
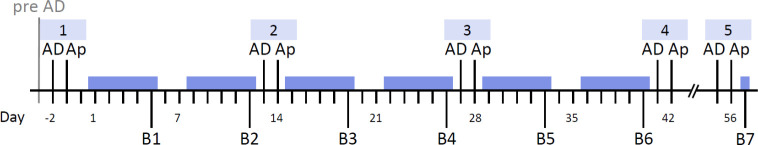
Experimental design. pre AD, pre-experimental avoidance distance test; AD, avoidance distance test; Ap, approach test; B1–7, behavioural observations. Treatment days are marked with a blue bar. Test numbers are given above AD and Ap indications, highlighted in light blue. For B7, the animals were treated as during the treatment period.

From each herd, 18 German Holstein cows with an avoidance distance (see test description, section 2.5) of at least 0.3 m were each assigned to one of three treatments randomly, but balanced for category of lactation (1, first lactation; 2, second or third lactation; 3, fourth lactation or higher), lactation day, horn status and avoidance distance. No cows with calf at foot were involved in the experiment. The mean lactation number of the experimental cows was 3 (min. 1 –max. 9), and at the beginning of the treatment period (day 1 in Fig 1), the experimental cows were on average in milk since 125 (10–244) days. The cows remained in their respective herd during the entire study period. The study was registered and approved by the responsible authority, the Ministry of Energy, Agriculture, the Environment, Nature and Digitalization in Schleswig-Holstein (file number V244-1713/2019).

### 2.2 Experimental design

All treatments and tests were conducted in the barn. The handler stroked the cows in the first treatment and talked to them in a gentle voice while they were restrained in the feeding rack (LOCK, n = 12). The cows in the second treatment experienced the presence of the handler, talking in a gentle voice and, if possible, stroking while unrestrained and able to move freely in the barn (FREE, n = 12). The third treatment group comprised control cows, which were not stroked or talked to at all (CON, n = 12).

The treatments were applied on 10 days out of each 14-day interval over the course of 6 weeks (Fig 1). For testing the animal-human relationship, avoidance distance tests and approach tests [5] were conducted before the start of the treatment period (test 1) and after every 14-day interval (tests 2–4), as well as 2 weeks after the end of the treatment period (test 5). The cows’ behaviour during the treatment was recorded on video once a week and additionally 2 weeks after the end of the treatment period to assess the reactions after 2 weeks without treatment.

### 2.3 Experimental treatment

Two handlers (both female, brown hair, green overall; height person A: 1.80 m, person B: 1.63 m) applied the treatments, one handler always treating the same herd. Before the start of the experiment, both handlers moved through both herds, talking in a gentle voice to the animals, so that they were not completely unfamiliar to the animals when they conducted the behavioural tests. This procedure was repeated on the afternoon before every testing day.

The handlers fed a small amount of concentrate (170 g) to all experimental animals while they were restrained in the feeding rack after the morning milking during the first 5 days of treatment to facilitate the establishment of a positive relationship. Also the CON animals received the concentrate to make sure that any differences between CON and LOCK or FREE were due to the treatment and not to the provision of feed. The CON animals did not experience any additional experimental treatment and were only subjected to the approach and avoidance tests.

The treatment of LOCK animals took place after the morning milking, when all animals were restrained in the feeding rack. On treatment days, all cows remained in the feeding rack during the duration of the treatment of the LOCK group, except for animals standing next to LOCK animals, which were released to provide space for the handler (these could also be FREE, CON or other LOCK animals). The treatment started only when the animal had finished feeding or had fed for at least 30 min. The handler addressed the animal verbally before establishing physical contact at the back or shoulder. She approached from the right side, as the cows were more used to physical contact starting at the right side from regular ratings of body condition score and injuries. The handler stroked the cow for 3 min while talking to her in a gentle voice. She reacted to the animal’s expressive behaviour (e.g. neck stretching, presentation of specific body parts) and stroked the parts of the head/neck region the cow seemed to prefer [22]. Stroking speed was 40–60 strokes/min as in previous studies [10,15], and the handlers had standardized the applied pressure between themselves. They wore rubber gloves with a rough surface (LUX paver’s gloves, OBI Bau-und Heimwerkermärkte, Vienna, Austria) during the treatments. If an animal showed defensive behaviour (e.g. head tossing, moving away from the handler), the handler continued stroking at the withers or shoulder until she could stroke the head/neck region again. Once all the LOCK animals in one feeding group were treated, the handler opened the feeding rack and animals of all treatments were free to leave the feeding area. The stroking treatment was started alternatingly in the early-lactation and in the late-lactation feeding group of each herd.

The treatment of FREE animals took place in the morning (approximately between 09:00h and 11:30h) when the animals were free to move in the barn. The handler approached FREE animals in a non-threatening way (no excessive body tension, slow movements, avoiding eye contact), paying attention to the body language of the animal and aiming to stop the approach before the animal showed an overt avoidance reaction. When the animals could be approached without the handler eliciting avoidance behaviour, or even sought contact, the handler slowly started to touch and stroke them, similar to the LOCK animals. The body region that was touched first depended on the behaviour of the animal: if the cow approached the handler, she touched the head first; if the cow did not show approach behaviour, the handler touched the back or shoulder first, as in the LOCK animals. The treatment was conducted for 3 min per day; the time counted from the first approach of the handler and was paused if the treatment was stopped due to the animal moving away or showing threatening behaviour towards the handler, to be started again with the next approach.

### 2.4 Observations of behaviour during treatment

During every fifth treatment, the animals’ behaviour was recorded by a technician using an HD Camcorder (SONY HDR-CX730, Weybridge, UK) for later analysis with the software BORIS [version 7.8.2; 23] according to an ethogram (S1 Table). While it was not possible to blind the observers with regard to the treatment, the order of observations was randomized so that the observers did not know whether they observed a treatment of the beginning or end of the experimental phase.

### 2.5 Avoidance distance tests

Avoidance distance tests in the barn [9,24] were conducted by both persons on both herds, so that either herd was tested twice on any given testing day–first by the handler, a familiar person that was not blinded towards treatment allocation, and after that by the handler of the other herd, a less familiar (in the following: “unfamiliar”) person that was blinded. All cows were tested in the morning, the tests starting after the morning milking and finishing at noon. If a cow could not be tested in the morning, the test was done in the afternoon or, on one occasion, on the next day before the handlers conducted the approach tests, but not directly before the approach test. If an animal stood in the alley in a suitable position (e.g. its way of retreat should not be blocked), the test person started from a distance of 3 m and approached the animal from the front at a speed of 1 step/s. One arm was extended in front of her at an angle of about 45°, with the back of the hand pointing forwards. The distance between the animal's muzzle and the test person’s hand was estimated in steps of 10 cm at the moment when the cow avoided the test person by taking a step or withdrawing the head. If the cow did not avoid her, she touched the cow's nose with the back of her hand. If the cow was touched, an avoidance distance of 0 cm was assigned, and the handler tried to stroke the cow’s cheek for 5 s. The touch score was recorded as ‘avoidance at touching’, ‘possible to touch (but not stroke)’ and ‘stroking possible + duration in s’. If an experimental cow reacted to the test of a neighbouring animal, she was not tested directly afterwards but at a later point in time.

Prior to the start of the experimental phase, there was an additional avoidance distance test, conducted by the future handler of each herd, that was not evaluated. The avoidance distance can decrease from the first to the second test, probably due to habituation to the testing procedure [9], and by ensuring that every animal was tested at least once before the data collection started, we aimed to diminish this effect.

### 2.6 Approach test

The approach test in the barn was conducted by the handler of the respective herd (the familiar person) in the morning of the day after the avoidance distance test. The test was started when a cow was standing in an alley in a suitable position, i.e. standing in a way that she could see the test person and that her way was not blocked, e.g. by other animals or the cubicles. The test person went to a position at 3 m distance from the cow’s head and remained there passively without encouraging contact for the test duration of 3 min, looking at her but not directly into her eyes. If the cow approached until establishing contact, the test person waited for 10 s after she established contact and started to stroke her until the test was terminated because the 3 min had passed or the animal walked away. If the cow moved away for more than 3 m (without having approached to contact) or started feeding, using the brush or interacting with another animal before the 3 min had passed, the test was terminated ahead of time and repeated some time later, as well as when another animal interrupted the test. This procedure was adopted in order to reduce the influence of competing motivations. There were no more than three test attempts per animal per testing day. If three attempts were unsuccessful, the maximal latency (180 s) was assigned.

### 2.7 Statistical analysis

The effect size could not be reliably estimated for sample size calculations, as there were no comparable data in the literature. We nevertheless carried out a power calculation for an unpaired t-test on avoidance distances with the software G*Power, version 3.1.9.2 [25], assuming a difference of one standard deviation between two of the groups and requiring a power of 80% and a significance level of α = 0.05, leading to a necessary sample size of n = 12 for these groups.

Data were analysed and presented graphically using the statistics environment R, version 3.5.2 [26]. Differences, main effects and interactions with P ≤ 0.05 are referred to as significant, with P ≤ 0.1 as a tendency. Statistics were calculated using the individual animal as statistical unit. Observations of animals that were obviously lame on the day of testing (three observations in tests 4 and 5, two animals) were removed from the data set, and there were no data for one animal’s third approach test and another animal’s first avoidance distance test with the unfamiliar person, resulting in a total sample size of 175 observations (59 CON, 56 LOCK, 60 FREE).

The seven variables derived from the behavioural tests–avoidance distance towards the familiar and unfamiliar person, touch score in the avoidance distance tests with the familiar and unfamiliar person, latencies to approach into a perimeter of 1 m around the familiar person and to contact, duration of contact with the familiar person–were reduced to one score using principal component analysis (PCA; function *prcomp*). The first resulting principal component (Table 1) was well interpretable, with all variables indicating acceptance or seeking of physical contact with the test person loading positively and all variables indicating a motivation to keep a distance to the person or a lack of motivation to approach loading negatively. Thus we denote the values of the first component as “relationship score”, with higher values indicating a stronger motivation for or acceptance of physical contact with the person and lower values indicating a stronger avoidance of the person.

**Table 1:** Eigenvalue of the first principal component (PC1, denoted “relationship”) derived by the PCA on the outcomes of the behavioural tests, variance explained, and loadings of the behavioural variables.

	PC1
Eigenvalue	1.97
Variance explained	0.56
**Loadings**	
AD familiar	-0.34
Touch score familiar	0.39
AD unfamiliar	-0.35
Touch score unfamiliar	0.39
Latency to 1m	-0.41
Latency to contact	-0.40
Duration of contact	0.37

We used the relationship score as our response in a linear mixed model (LMM) with the package *lme4* [27], with treatment and test number and their interaction as fixed factors and the animal nested in the herd as random factor to take into account the repeated measures. The distributions of residuals and homogeneity of variance were checked visually, and to fulfil model assumptions, the relationship score was log-transformed after adding a value of 2.3 to obtain positive values.

To investigate the interaction between treatment and test number in more detail, we calculated the change in the relationship score from test 1 to test 4 and from test 1 to test 5, as the most obvious effects of the treatment were expected for tests 4 and 5. We evaluated both resulting variables with LMMs including the same random effects as the main model and treatment as the only fixed effect, and corrected the results for multiple testing using false discovery rate control (FDRC) [28]. Subsequently, we calculated pairwise comparisons with Tukey correction using the package *emmeans* [29]. For detailed model descriptions, see S2 Table. The behaviour during the treatment is presented descriptively.

## 3 Results

### 3.1 Behaviour during the treatment

**Fig 2:**
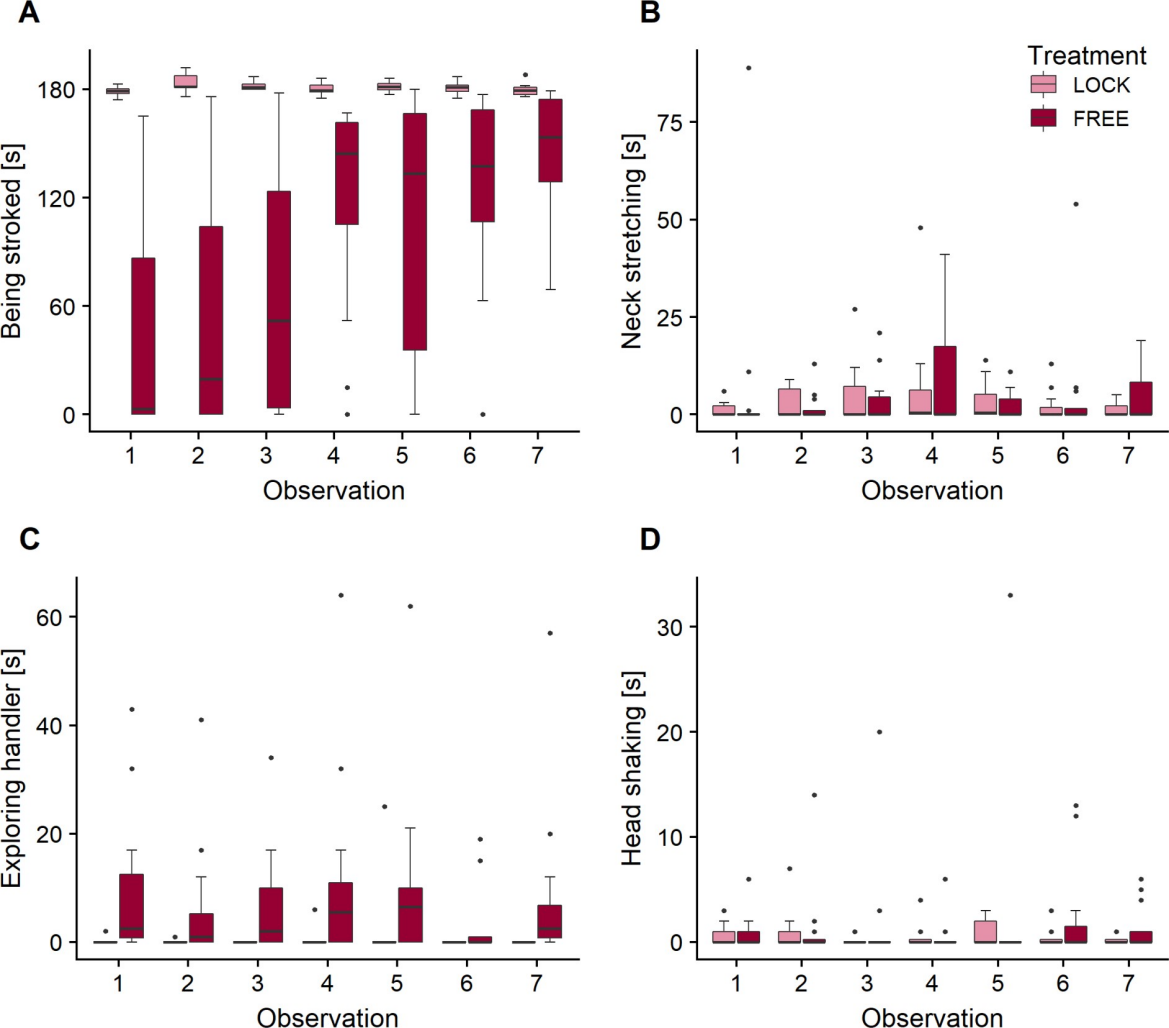
Behaviours shown by cows in the LOCK and FREE groups during gentle interactions. A) Being stroked, B) neck stretching, C) exploring the handler, D) head shaking. The FREE group (n = 12) experienced gentle interactions with a person while free in the barn, the LOCK group (n = 12) while restrained in the feeding rack. The treatment period comprised 6 weeks, with a total of 30 treatment days; behavioural observation took place during each fifth treatment. Observation 7 was not part of the regular treatment but served as a test situation in order to assess the animals’ reactions after 2 weeks without gentle interactions.

The behaviour during the treatment was not analysed using statistical tests, but evaluated on the descriptive level. Over the course of the experimental period, the FREE cows accepted increasingly longer durations of stroking (Fig 2A). In observation 1 the median stroking duration was 3 s (first–third quartile: 0–86 s), and the largest increase was between observation 3 (median 52 s, 4–124 s) and observation 4 (144 s, 105–162 s). After observation 4, the duration of stroking stayed relatively stable until it reached its maximum in observation 7, two weeks after the treatment period ended (154 s, 129–174 s). In the LOCK treatment, the median duration of stroking was around 180 s throughout the whole experimental phase, reflecting that LOCK animals could not avoid stroking.

The duration of neck stretching (Fig 2B) was very low in both groups, the median staying at 0 s throughout the experimental phase. In the LOCK treatment group, the third quartile increased from observation 1 (2 s) to observation 2 (6 s) and stabilized at that level before decreasing again in observation 6 (2 s). In the FREE group, the third quartile increased more slowly, peaked at observation 4 (18 s) and decreased thereafter, only increasing again slightly in observation 7, two weeks after the end of the treatment period (8 s).

Some behaviours, especially contact-seeking behaviours such as exploration (Fig 2C), rubbing or licking of the handler, occurred numerically more often in FREE than in LOCK (overall median & Q3: FREE 2 s, 10 s; LOCK 0 s, 0 s). Behaviours possibly indicating a negative perception occurred rarely (head shaking, Fig 2D; overall frequencies for FREE, as there was no occurrence in LOCK: walking away 8, threatening 7). Graphs of behaviours not depicted in Fig 2 are included in the supporting information (S1 Fig).

### 3.2 Avoidance distance and approach tests

**Fig 3:**
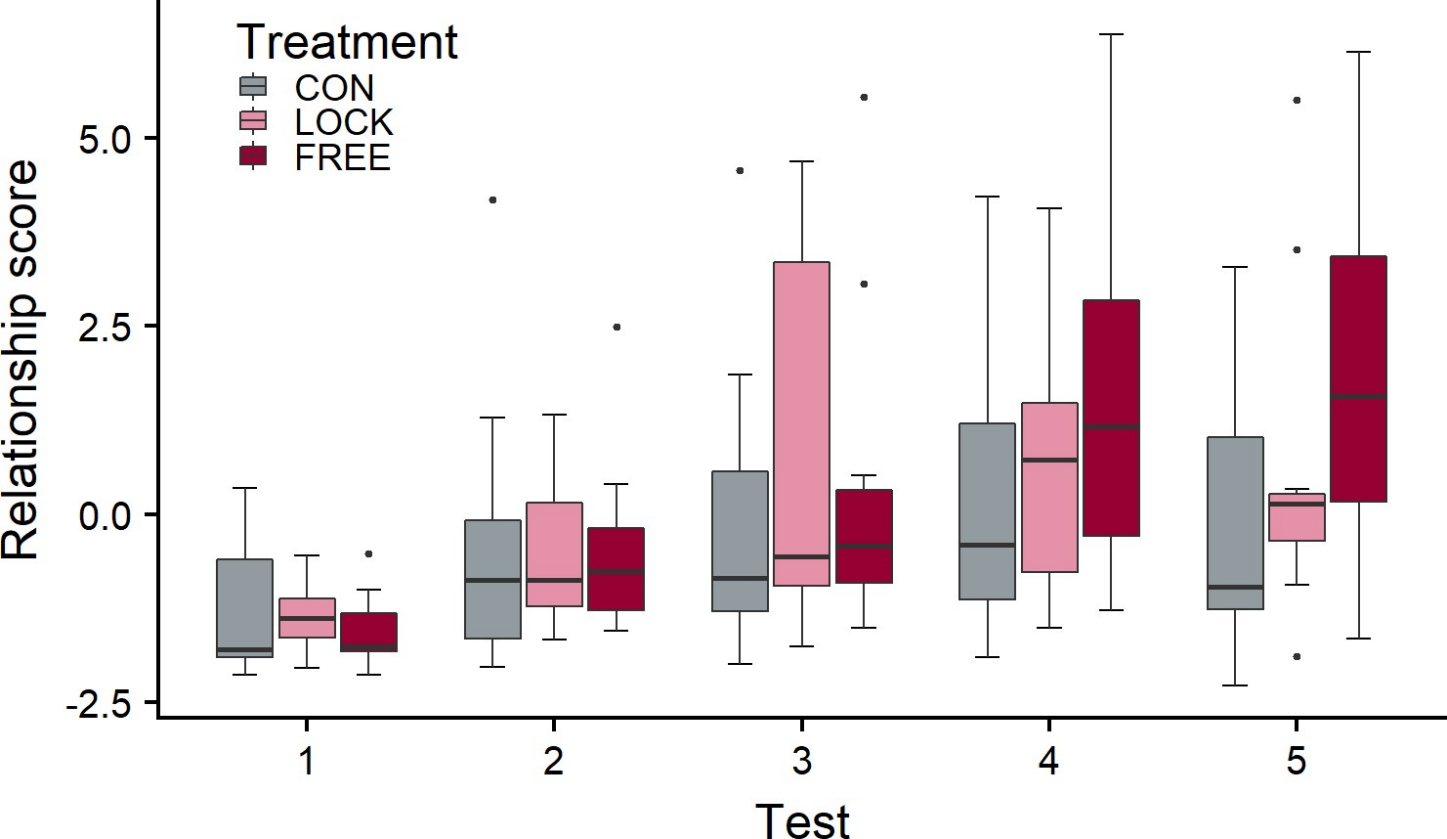
The relationship score in the three treatment groups over the course of the study. The FREE group experienced gentle interactions with a person while free in the barn, the LOCK group while restrained in the feeding rack; the CON group did not experience gentle interactions. The treatment period comprised 6 weeks with a total of 30 treatment days between tests 1 and 4; during the 2 weeks between tests 4 and 5, no treatment took place. LMM: test number p = 0.008, test number × treatment p = 0.014; n = 12 per treatment group and test number, except for test 1 n_CON_ = 11, test 3 n_LOCK_ = 10, test 4 n_LOCK_ = 11, test 5 n_LOCK_ = 11.

The results of the individual behavioural variables are depicted in S2 Fig. The relationship score increased over the course of the experiment in all groups (Fig 3; main effect of test number: χ^2^ = 7.0, df = 1, p = 0.008), and there was a significant interaction of test number and treatment (χ^2^ = 8.5, df = 2, p = 0.014), with the relationship score being highest in FREE, lowest in CON and intermediate in LOCK at the end of the experimental period.

**Fig 4:**
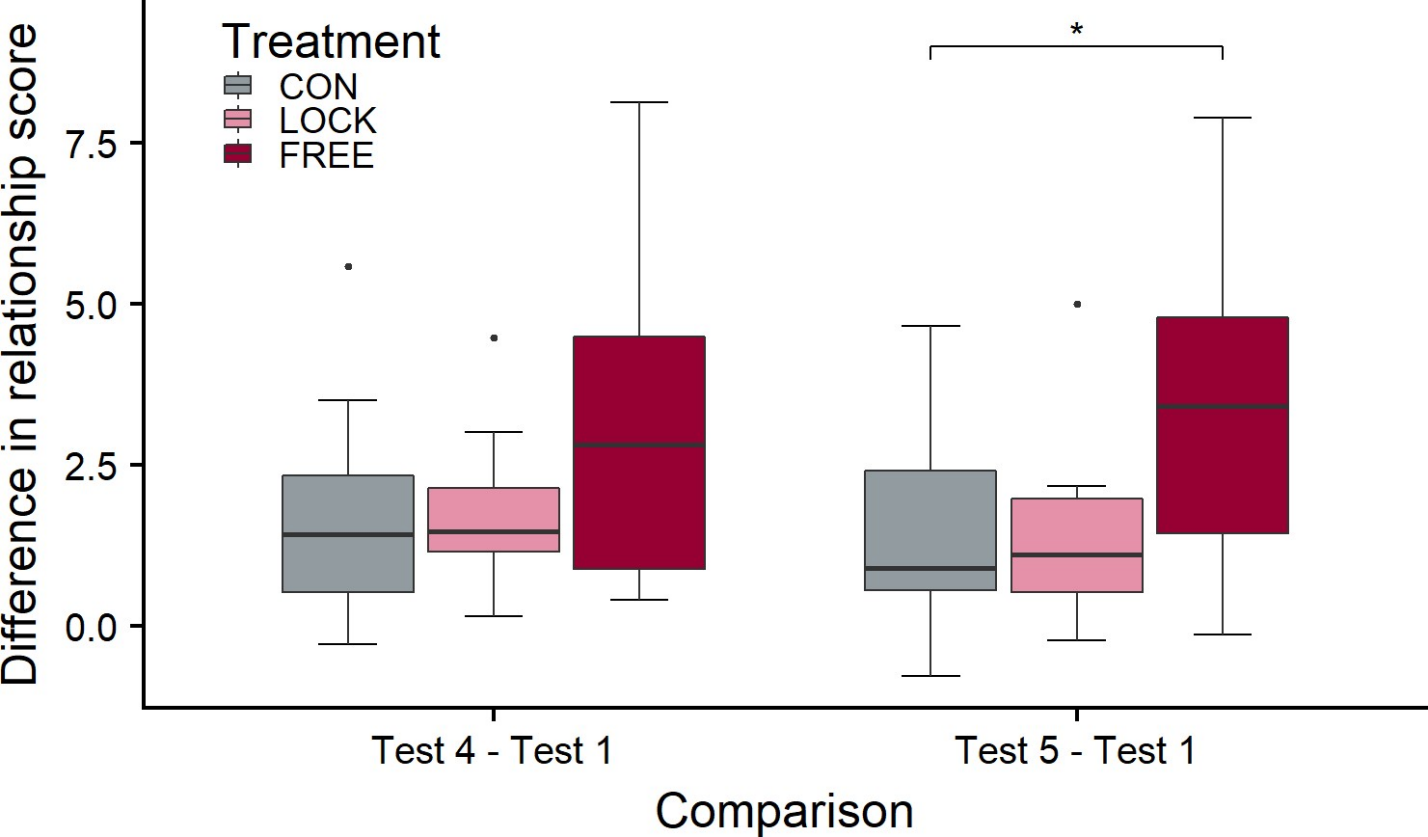
Change in the relationship score from test 1 to test 4 and from test 1 to test 5 in the three treatment groups. The FREE group experienced gentle interactions with a person while free in the barn, the LOCK group while restrained in the feeding rack; the CON group did not experience gentle interactions. Test 4 took place after the treatment period of 6 weeks with a total of 30 treatment days, test 5 2 weeks later. No treatment took place between tests 4 and 5. LMM: treatment p < 0.1 for test 5, ns for test 4; *: p < 0.05 for pairwise comparison; n_CON_ = 11, n_LOCK_ = 11, n_FREE_ = 12.

The change in the relationship score from test 1 to test 4 (Fig 4) was not significantly different between groups (χ^2^ = 14.1, df = 2, p = 0.19), but the comparison of the change between tests 1 and 5 revealed a trend towards a difference between groups (χ^2^ = 27.6, df = 2, p < 0.1 after FDRC). The increase in the relationship score from test 1 to test 5 was significantly higher in FREE than in CON (df = 31, t = -2.55, p = 0.041), whereas CON and LOCK (df = 31, t = -0.52, p = 0.86) and LOCK and FREE (df = 31, t = -2.02, p = 0.12) did not differ significantly.

## 4 Discussion

The main finding was a general reduction of avoidance distance over the course of the treatment period, which was strongest in FREE, followed by LOCK. It was paralleled by an increase in approach behaviour, which again was most pronounced in FREE animals, followed by LOCK, but not statistically significant.

### 4.1 Behaviour during the treatment

The descriptive evaluation of the behavioural observations revealed that FREE animals accepted increasingly longer durations of stroking over the course of the experiment, which indicates a successful process of habituation and, perhaps, positive reinforcement due to a positive perception of the interactions. The biggest increase was between observation 3 (after 15 treatments) to observation 4 (after 20 treatments), indicating that more than 15 treatments (45 min) were necessary to improve the animal-human relationship to a degree where most cows could be stroked for more than half of the treatment time. The treatment was not equally accepted by all FREE animals: even in observation 6, after 87 min of treatment, one cow did not accept stroking. The longer durations of stroking in observation 7, two weeks after the end of the treatment period, might indicate a rebound effect: the cows’ increased acceptance of stroking after they lacked the opportunity to engage in positive human-animal interactions for two weeks might indicate an increased motivation for gentle tactile stimulation. It is noteworthy that in this last observation, all FREE cows accepted stroking for at least 1 min, indicating that there were longer-term effects of the treatment, improving the animal-human relationship further even after the end of the treatment period, at least in individual animals.

During stroking in both treatments, some cows showed neck stretching, although with relatively low durations. As this behaviour is shown during actively solicited social grooming [8,30,31] and stroking by humans [14], it is interpreted as a sign of enjoyment [14,15,32] and thus indicates a positive perception of the stroking treatment. As could be expected, considering the lower acceptance of stroking in FREE animals during the first three observations, the LOCK group showed more neck stretching during the first three observations. In observation 4, when the acceptance of stroking was higher, more neck stretching occurred in the FREE group as well. This pattern might indicate that once the animals can be touched, the transition to a positive perception of the interaction occurs relatively quickly.

With regard to the other behaviours, it has to be considered that most of them could be more easily expressed by the FREE animals, as the possibilities of movement of the LOCK animals were restricted. However, the numerically higher occurrence of some behaviours, such as exploration of the handler or walking away, might indicate that the cows indeed used their behavioural freedom to control actively the intensity of the interaction with the handler or to avoid the interaction.

### 4.2 Avoidance distance and approach tests

In line with our hypothesis, the relationship score increased in the three treatment groups to different degrees. We predicted that this increase should be visible earlier in LOCK than in FREE animals due to the increased opportunity to habituate to close human presence and physical contact in this context, also allowing positive reinforcement at an earlier time point during the study. However, the increase from test 1 to test 2 was similar in all three groups. In test 4, the median relationship score was lowest in the CON and highest in the FREE group, with the score of the LOCK animals being more similar to that of FREE than of CON animals, but the increase from baseline (test 1) to test 4 was not significantly different between groups. In contrast, the relationship score of the FREE animals increased more strongly from test 1 to test 5 than in CON animals, with that of LOCK animals being intermediate and not significantly different from the other groups’ scores. This result is in line with our expectation that the effects of the treatment on avoidance and approach behaviour would be more sustained and thus more pronounced in FREE than in LOCK animals two weeks after the end of the treatment. Although it was unexpected that the relationship score increased even after the end of the treatment period, the finding confirms an earlier study in tied dairy cows, where the avoidance reaction towards the handler was lower 4 weeks after the end of the treatment (3 weeks of stroking the ventral neck) compared with the test directly after the treatment [6].

It seems thus that the increased controllability of the situation as perceived by cows that are able to move freely during gentle interactions with humans has a beneficial influence on the improvement of their relationship with humans, as already hypothesized by Le Neindre et al. [20], and that this effect outweighs the benefits of restraint regarding a faster habituation of the animals. It is possible that the first treatments were perceived as aversive by the LOCK animals because all experimental animals had a suboptimal relationship with humans, as indicated by their moderate to high avoidance distances. In this case, close physical contact with humans might be perceived negatively by the animals until habituation sets in, and this negative affective state will be exacerbated by the lack of possibilities to avoid the treatment. In contrast, even the first treatments of the FREE animals probably had a positive component, as the animals could satisfy their curiosity, and the negative component was most likely smaller than in the LOCK group, as the animals were able to control to which extent they accepted the contact with the person. Correspondingly, they had a higher level of agency, which again evokes positive emotions [19]. In addition, the interactions were potentially more mutual in this situation, as it was easier for the animals to explore and lick the person as well as to present specific body parts they preferred to have stroked. In the LOCK animals, a true, mutual interaction was much more difficult, as the restraint not only prevented the animals from avoiding the treatment but also hampered active participation, possibly reducing the positive perception of the interaction.

The relationship score of the CON group increased over the study period as well, although to a lower degree. This might seem surprising because they did not experience gentle interactions with the handler, but there are several mechanisms that can explain the result. The increase from test 1 to test 2 might have been influenced by the feeding of concentrate by the handlers on the first five days [33–35]. In general, CON animals probably lost some of their fear towards humans by the frequent presence of the handlers in the barn [36–38]. Through a process of habituation, they might have started to learn that the person poses no threat to them (the person imposing neither negative nor positive interactions), which led to an improvement of their relationship with humans. Close human presence did not reduce avoidance reactions in tied dairy cattle in contrast to stroking [6]; however, as the cows were tied in that study, they had no control over the distance to the human during the ‘presence’ treatment, in contrast to our CON cows, which were able to keep a distance to the person. A third mechanism might be social transmission [39]: the CON cows witnessed the interactions with the LOCK and FREE cows and their reactions and consequently adapted their behaviour accordingly. While social transmission has not been studied thoroughly in cattle, there are some studies indicating that cows are able to adapt their behaviour according to the behaviour of conspecifics [40,41]. Regarding the animal-human relationship, gentle interactions between handlers and tethered cows led to a decreased distance to handlers not only in the treated cows but also in neighbouring cows that could observe the treatment, and the distance the observing cows kept was correlated with the distance the treated cows kept [18].

Another point that needs to be addressed is the actual duration of gentle tactile contact in the LOCK and the FREE treatments. Our experiment was not designed to investigate primarily the effect of the level of control over the situation as perceived by the animal. To this purpose, we would have needed to record the duration of vocal and tactile contact with the FREE animals and also the distance kept by the animals and then to treat matched LOCK animals in the same way, as yoked controls [42]. Instead, we opted for an approach that would be more relevant for practice, answering the question whether gentle interactions with or without restraint were more effective at improving the animal-human relationship under the condition that the farmer invests the same amount of time in interacting with the animals. The FREE animals were thus stroked for a shorter time, in total, than the LOCK animals, but still showed a stronger improvement of their relationship with humans. Thus, we can conclude that the duration of the tactile interaction is not the main factor influencing the effectiveness of gentle human-cow interactions. Other characteristics have to play a role, and one of the characteristics that differ clearly between the situations is animal agency or perceived controllability of the situation.

## 5 Conclusion

Interacting gently with free-moving dairy cows in the barn improved the animal-human relationship to a higher degree than interactions during restraint in the feeding rack. This might be due to a stronger sense of control over the situation, and thus agency, and the ability to avoid or intensify the contact with the person according to the animal’s motivation, potentially leading to a more pleasurable experience. Thus, we recommend that gentle interactions with dairy cows should take place while they are unrestrained, if possible.

## Supporting information

S1 Fig Behaviours shown by cows in the LOCK and FREE groups during gentle interactions. (DOCX)

S2 Fig Results of the avoidance distance and approach tests. (DOCX)

S1 Table Ethogram for behaviours coded during gentle interactions. (DOCX)

S2 Table Model descriptions. (DOCX)

S1 File Raw data. (XLSX)
