# Supplementary material for: Gentle interactions with restrained and free-moving cows: Effects on the improvement of the animal-human relationship
Source: PLoS One. 2020 Nov 23;15(11):e0242873. doi: 10.1371/journal.pone.0242873 (PMC7682860; doi:10.1371/journal.pone.0242873)
Supplement: S1 Fig — (DOCX) [file pone.0242873.s001.docx]

**S1 Figures**


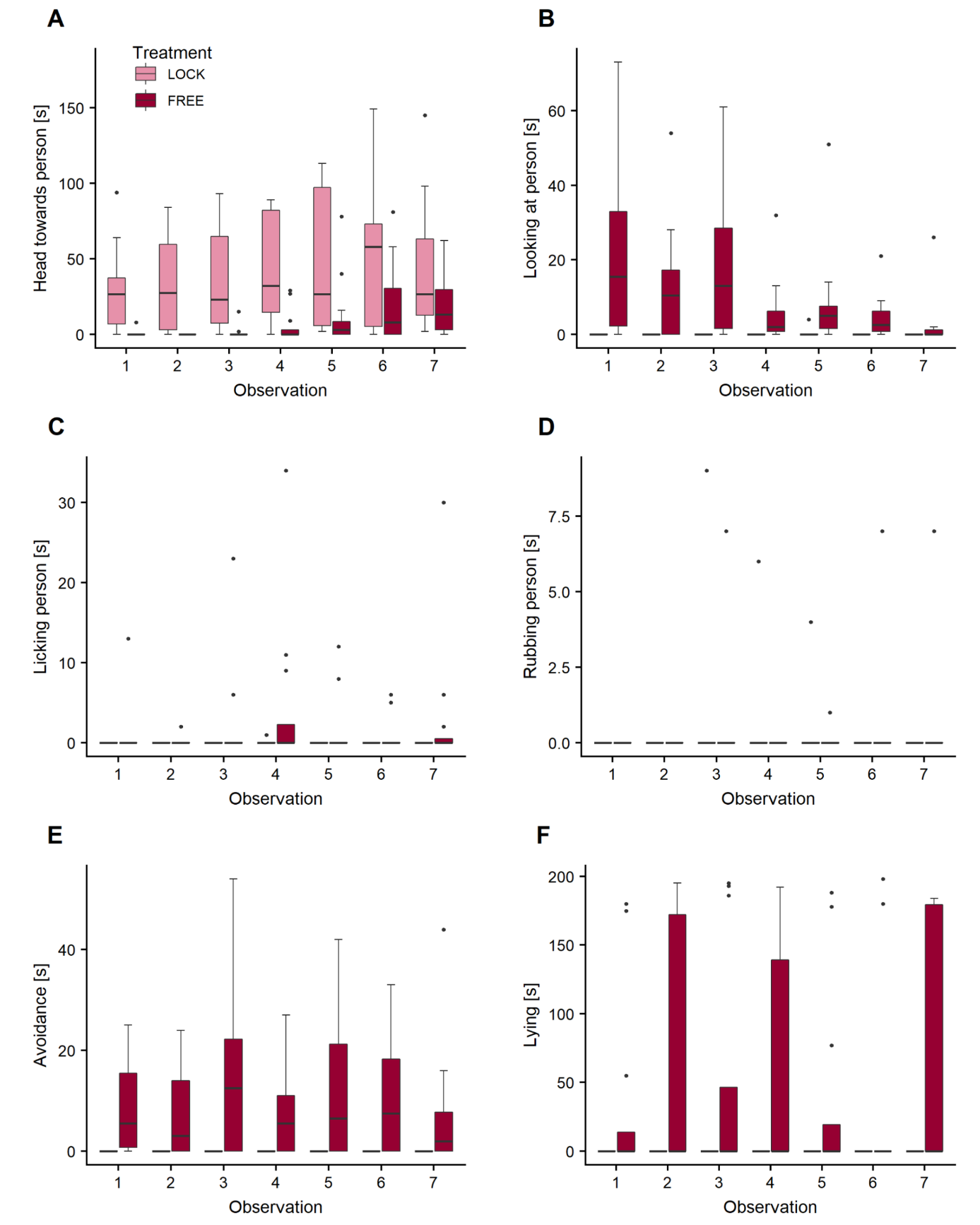


**Fig S1a. Durations of behaviours shown by cows in the LOCK and FREE groups during gentle interactions.** A) Head towards person, B) looking at person, C) licking person, D) rubbing person, E) avoidance, F) lying. The FREE group experienced gentle interactions with a person while free in the barn, the LOCK group while restrained in the feeding rack. The treatment period comprised 6 weeks, with a total of 30 treatment days; behavioural observation took place during each fifth treatment. Observation 7 was not part of the regular treatment but served as a test situation in order to assess the animals’ reactions after 2 weeks without gentle interactions. n = 24.


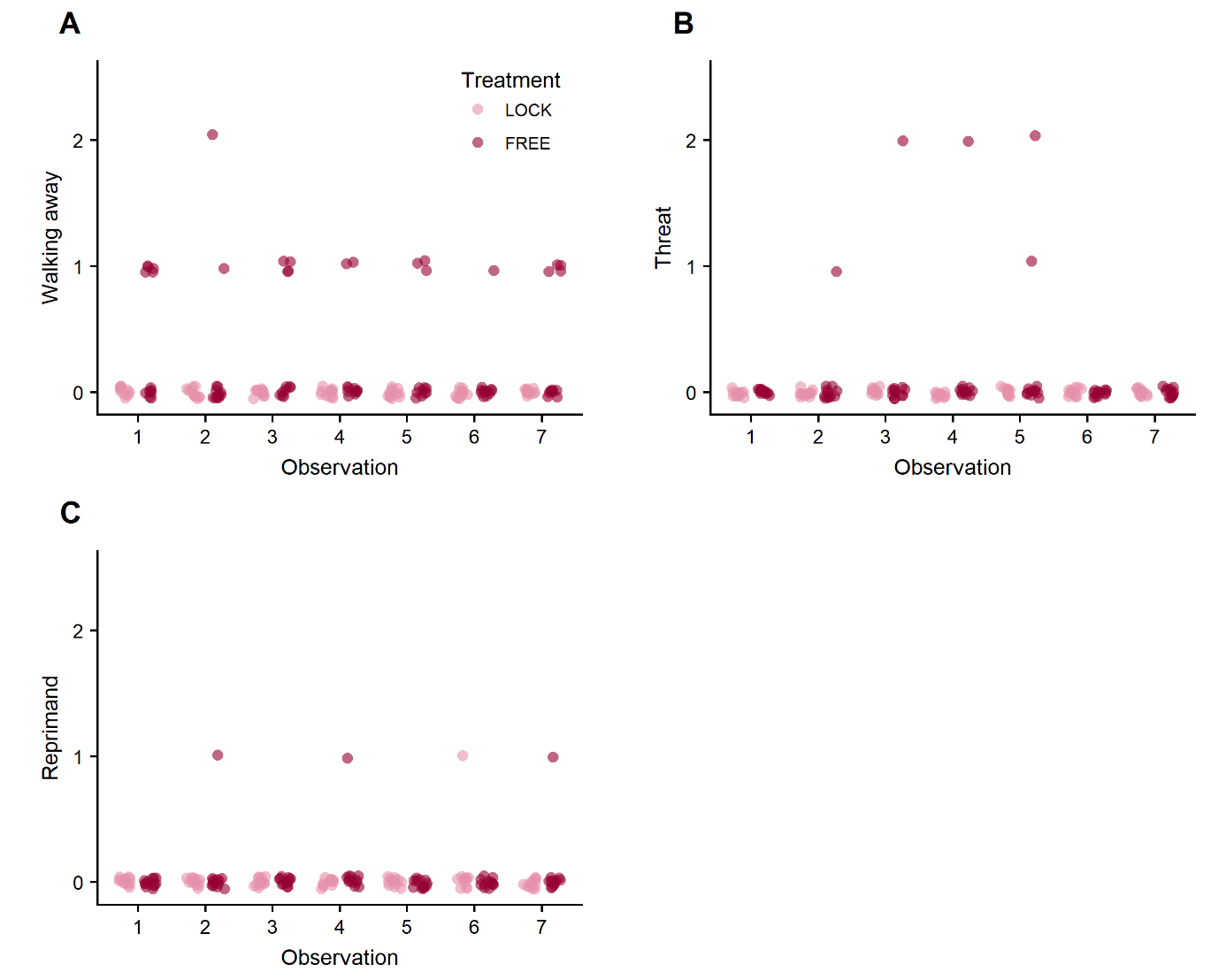


**Fig S1b. Frequencies of behaviours shown by cows in the LOCK and FREE groups during gentle interactions.** A) Walking away, B) threat, C) reprimand. Data points are jittered and transparent in order to facilitate distinguishing single points. The FREE group experienced gentle interactions with a person while free in the barn, the LOCK group while restrained in the feeding rack. The treatment period comprised 6 weeks, with a total of 30 treatment days; behavioural observation took place during each fifth treatment. Observation 7 was not part of the regular treatment but served as a test situation in order to assess the animals’ reactions after 2 weeks without gentle interactions. n = 24.
