# Supplementary material for: Gentle interactions with restrained and free-moving cows: Effects on the improvement of the animal-human relationship
Source: PLoS One. 2020 Nov 23;15(11):e0242873. doi: 10.1371/journal.pone.0242873 (PMC7682860; doi:10.1371/journal.pone.0242873)
Supplement: S2 Fig — (DOCX) [file pone.0242873.s002.docx]

**S2 Figures**


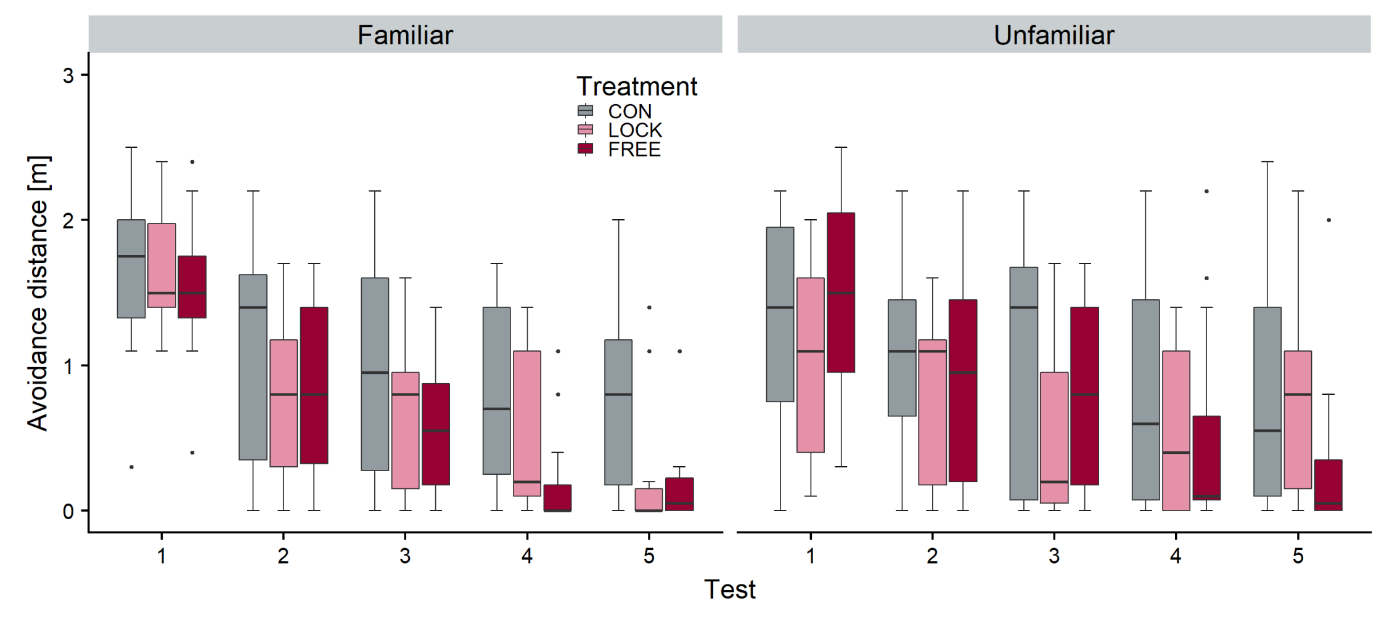


**Fig S2a. Avoidance distances of the three treatment groups over the course of the study in tests with a familiar and an unfamiliar person.** n = 36.


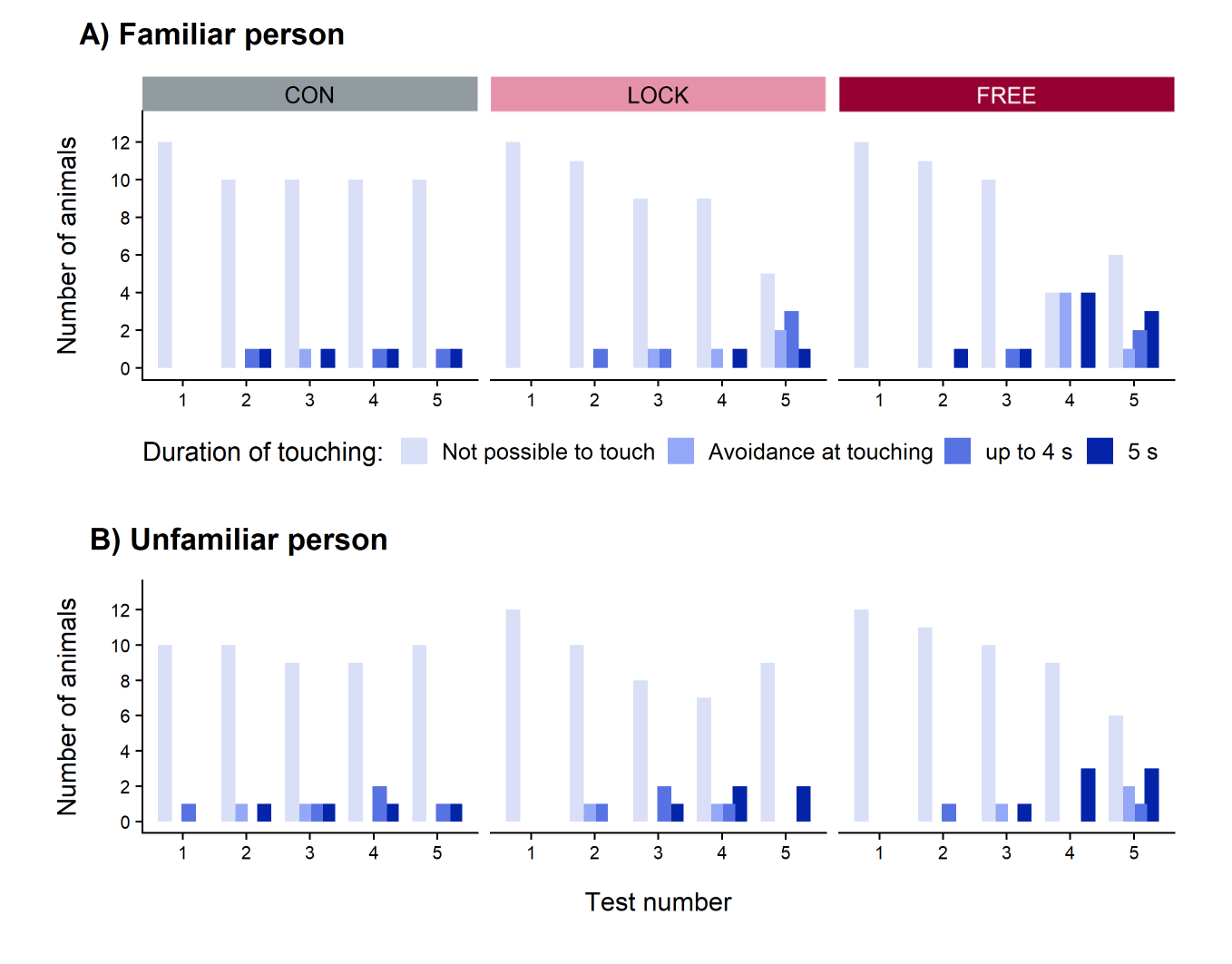


**Fig S2b. Touch score of the three treatment groups over the course of the study in tests with a familiar and an unfamiliar person.** n = 36.

**
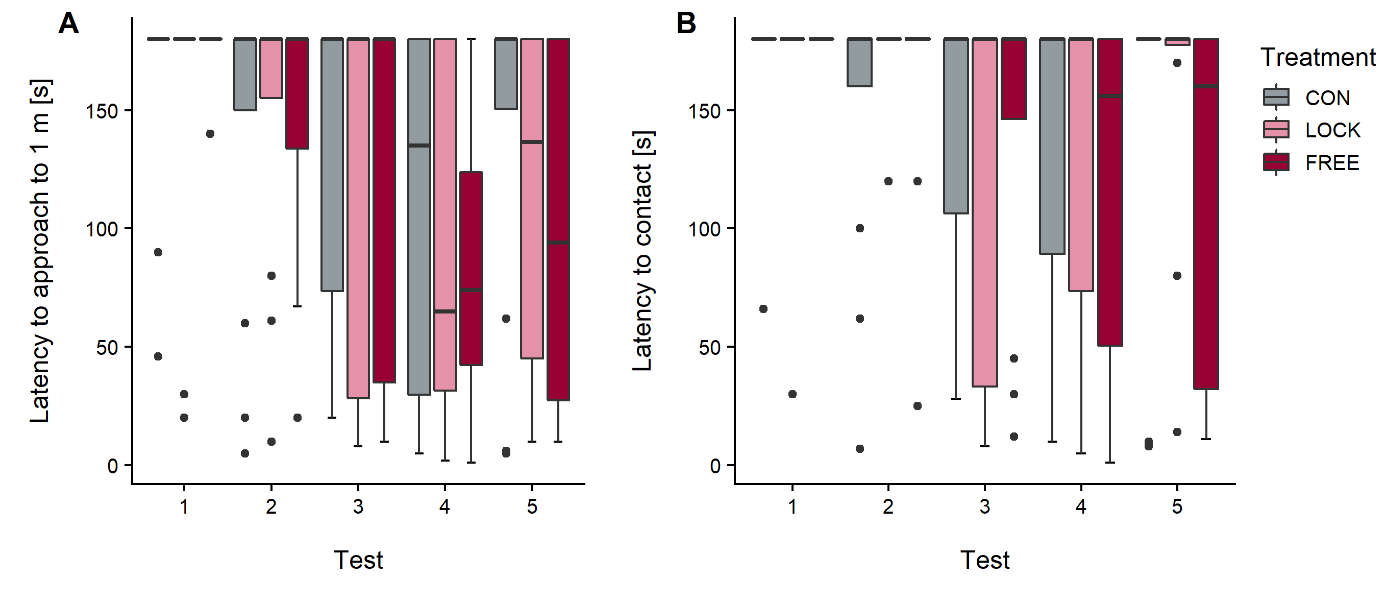
Fig S2c. Latency of animals in the three treatment groups to approach the familiar person up to 1 m of distance (A) or until physical contact (B) in the approach test over the course of the study.** n = 36.

**
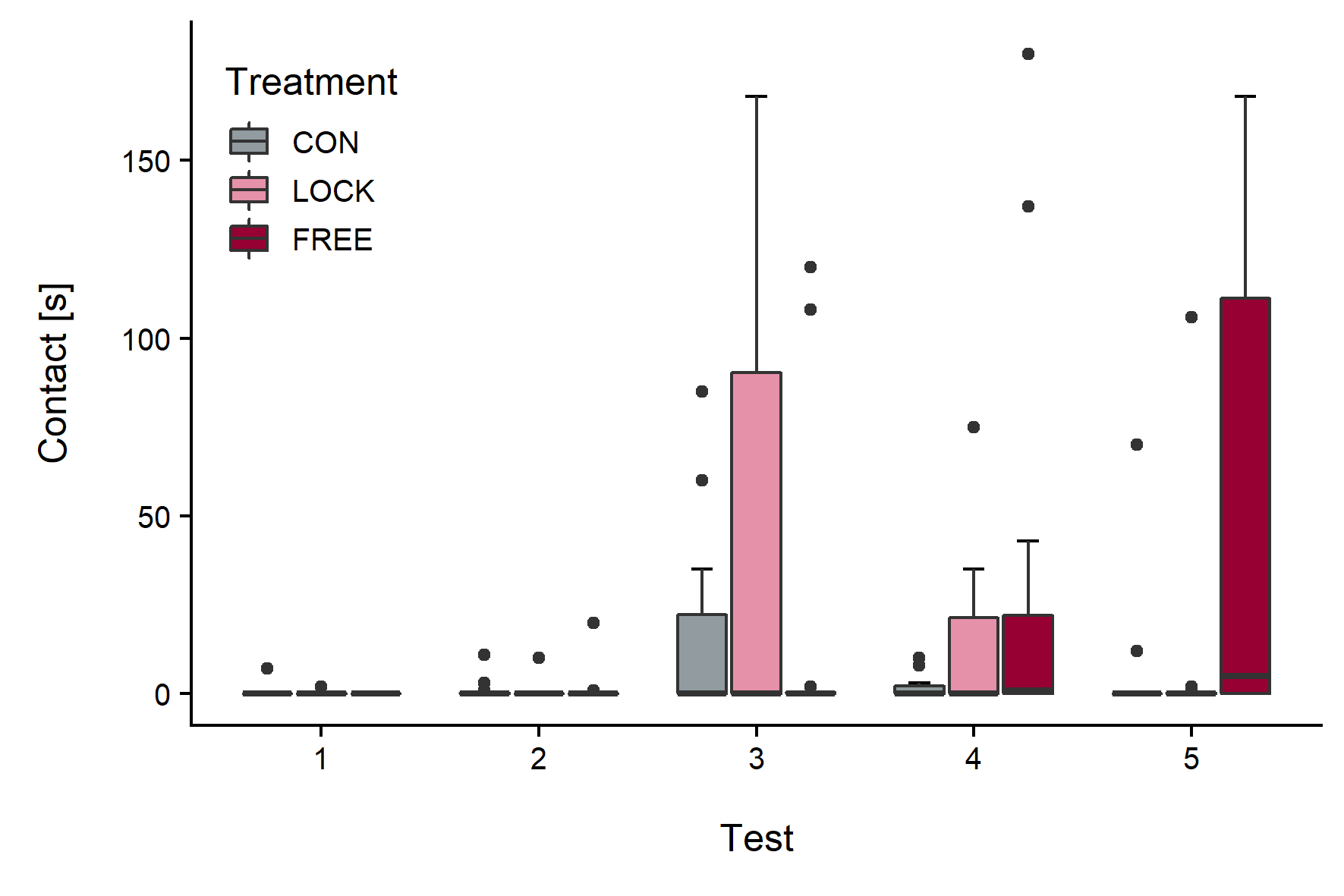
**

**Fig S2d. Duration of contact of animals in the three treatment groups with the familiar person in the approach test over the course of the study.** n = 36.
