# Supplementary material for: Gentle interactions with restrained and free-moving cows: Effects on the improvement of the animal-human relationship
Source: PLoS One. 2020 Nov 23;15(11):e0242873. doi: 10.1371/journal.pone.0242873 (PMC7682860; doi:10.1371/journal.pone.0242873)
Supplement: S1 Table — (DOCX) [file pone.0242873.s003.docx]

# **Table S1:** Ethogram for behaviours coded during gentle interactions.

| **Behaviour** | **Definition** | **Type** |
| --- | --- | --- |
| *Lying* | Sternal or lateral recumbency: sternum or flank are in contact with the ground and no weight is supported by any of the legs. | duration |
| *Standing* | Body position in which the limbs bear the bodyweight. | duration |
| *Avoidance* | The animal moves out of reach of the person without obvious external influences, but stays within a perimeter of 3 m. The behaviour ends as soon as the animal stops moving or leaves the perimeter of 3 m. | duration |
| *Walking away* | The animal walks away > 3 m without obvious external influences. | frequency |
| *Head shaking* | Rapid rotational head movements, often when the person is approaching or trying to touch the animal and often pulling the chin to the chest as part of the movement. | duration |
| *Threat* | The animal presents her forehead: The head is lowered, the animal’s nose drawn towards to her chest. The position is not counted as threat if it occurs in the context of play behaviour. Another form of threat is head-tossing: The animal throws its head sideways/backwards towards the threatened subject (human, conspecific). | frequency |
| *Head towards person* | The animal has its head turned towards the person, deviating more than 30° from a neutral head position (head in line with spine) while being stroked. | duration |
| *Looking towards person* | The animal directs its head (nose) towards the person while not being stroked. | duration |
| *Exploring person* | The animal moves its muzzle towards the person into a perimeter of 10 cm, muzzle pointing towards the person. The behaviour ends when the animal's muzzle does not point towards the person anymore or leaves the perimeter of 10 cm. | duration |
| *Rubbing person* | The animal touches the person and moves the touching body part while in contact with the person. The behaviour ends when the contact between the animal and the person is interrupted for at least 3 s. | duration |
| *Licking person* | The animal’s tongue touches the person or an object connected with her (e.g. the clipboard), or the typical up-and-down or forward-backward head movement is shown (if tongue is not visible). | duration |
| *Neck stretching* | Positioning the neck and head actively in an outstretched line. Neck stretching was not recorded if the position was attained during feeding, rubbing, licking or rubbing or licking of the person or during exploration (= if an elongated neck was necessary to explore the object/person). If neck stretching was shown already before rubbing, licking or rubbing or licking of the person, it was recorded as long as the neck was in an outstretched line. | duration |
| *Head play* | Up-and-down head movements, often while the animal is oriented towards the person; often with the poll directed forwards/chin pulled to the chest; can look like rubbing without establishing contact, but can also include physical contact; part of the movement often slightly rotational, not straight. | frequency |
| *Stroking* | The person actively touches the animal with hand or finger movements such as petting, scratching, stroking. The behaviour ends if interrupted for > 2 s. | duration |
| *Reprimand* | The person speaks with a loud voice or makes a sudden movement (such as stamping the foot, smacking the hand against experimenter’s thigh or slapping the animal with the hand, if other measures were unsuccessful). | frequency |
| *Not visible* | The video does not allow behavioural observation. |  |
