# Supplementary material for: Gentle interactions with restrained and free-moving cows: Effects on the improvement of the animal-human relationship
Source: PLoS One. 2020 Nov 23;15(11):e0242873. doi: 10.1371/journal.pone.0242873 (PMC7682860; doi:10.1371/journal.pone.0242873)
Supplement: S2 Table — (DOCX) [file pone.0242873.s004.docx]

# Table S2: Model results.

| **Relationship score** | | | | | | | |
| --- | --- | --- | --- | --- | --- | --- | --- |
|  | Coef | SE | CL_lower_ | CL_upper_ | χ^2^ | Df | P |
| (Intercept) | -0.280 | 0.215 | -0.720 | 0.163 | 1.691 | 1 | 0.193 |
| Treatment^1^ |  |  |  |  | 2.481 | 2 | 0.289 |
| LOCK | 0.103 | 0.301 | -0.510 | 0.708 |  |  |  |
| FREE | -0.346 | 0.300 | -0.955 | 0.257 |  |  |  |
| Test number | 0.164 | 0.062 | 0.028 | 0.296 | 7.013 | 1 | 0.008 |
| Treatment × test number^2^ |  |  |  |  | 8.532 | 2 | 0.014 |
| LOCK × test number | 0.078 | 0.087 | -0.096 | 0.259 |  |  |  |
| FREE × test number | 0.244 | 0.086 | 0.073 | 0.421 |  |  |  |

| **Change in relationship score from test 1 to test 4** | | | | | | | |
| --- | --- | --- | --- | --- | --- | --- | --- |
|  | Coef | SE | CL_lower_ | CL_upper_ | F | Df | P |
| (Intercept) | 1.647 | 0.605 | 0.414 | 2.880 | 7.423 | 1 | 0.010 |
| Treatment^1^ |  |  |  |  | 1.753 | 2 | 0.190 |
| LOCK | 0.274 | 0.855 | -1.470 | 2.017 |  |  |  |
| FREE | 1.464 | 0.837 | -0.243 | 3.171 |  |  |  |
| **Change in relationship score test 1 to test 5** | | | | | | | |
|  | Coef | SE | CL_lower_ | CL_upper_ | F | Df | P |
| (Intercept) | 1.350 | 0.585 | 0.157 | 2.543 | 5.324 | 1 | 0.028 |
| Treatment^1^ |  |  |  |  | 3.662 | 2 | 0.037 |
| LOCK | 0.430 | 0.827 | -1.258 | 2.117 |  |  |  |
| FREE | 2.064 | 0.810 | 0.412 | 3.716 |  |  |  |

LOCK, FREE: The FREE group experienced gentle interactions with a person while free in the barn, the LOCK group while restrained in the feeding rack.

Coef, coefficient; SE, standard error; CL, lower and upper confidence limit; Df, degrees of freedom.

^1^ Treatment was dummy coded with ‘CON’ (control cows, no gentle interactions) being the reference category; the indicated test refers to the overall effect of treatment.

^2^ The indicated test refers to the overall effect of the interaction.
